# Genetic diversity, virulence genotype and antimicrobial resistance of uropathogenic *Escherichia coli* (UPEC) isolated from sows

**DOI:** 10.1080/01652176.2018.1519321

**Published:** 2018-10-26

**Authors:** Maria G. Spindola, Marcos P. V. Cunha, Luisa Z. Moreno, Cristina R. Amigo, Ana P. S. Silva, Beatriz M. Parra, André P. Poor, Carolina H. de Oliveira, Barbara P. Perez, Terezinha Knöbl, Andrea M. Moreno

**Affiliations:** Faculdade de Medicina Veterinária e Zootecnia, Universidade de São Paulo, São Paulo, Brazil

**Keywords:** Swine, porcine, *Escherichia coli*, resistance, urinary tract infections, AFLP, virulence genes, phylogenetic group

## Abstract

**Background:** Urinary tract infections (UTI) cause severe losses to the swine industry worldwide and uropathogenic *Escherichia coli* (UPEC) are the main agent isolated from UTI in sows.

**Objective:** The aim of this study was to investigate the virulence genes, assess the phylogenetic background, clonal diversity, and the pattern of resistance to antimicrobials in 186 isolates of UPEC isolated from sows in Brazil.

**Materials and methods:** Urine samples from 300 sows of three herds with clinical signs from São Paulo State (Brazil) were screened for UTI; samples with suggestive results were submitted to bacterial isolation. *E. coli* strains isolated were characterized using disk diffusion technique, polymerase chain reaction and Single-enzyme amplification fragment length polymorphism (SE-AFLP).

**Results:** Virulence genes *foc*H and *pap*C were present in 78.5% and 58% of strains, respectively, followed by *cnf*1 (23.2%), *afa* (13.4%), *sfa* (11.3%), *iuc*D (6.9%), and *hly*A (1.6%). No clonal relatedness was found by SE-AFLP. A total of 98% of isolates (182/186) were multidrug resistant, and the highest levels of resistance were to sulfonamides, tetracycline, florfenicol, and ampicillin. Isolates were classified in phylogenetic group B1 (34.4%), followed by D (33.9%), E (30.1%) and A (1.6%).

**Conclusions:** The data obtained suggest that pigs from clinically affected herds may serve as a reservoir of uropathogenic and multidrug-resistant *E. coli* strains.

## 1. Introduction

Urinary tract infections cause severe losses to the swine industry worldwide, either due to therapeutic spending, early disposing of breeding sows, and acute death of severely affected sows (Drolet 2012). Several studies have shown that urinary tract infection (UTI) is a major cause of mortality and reduced life of sows (Abiven et al. 1998; Glock & Bilkei 2005; Sanz et al. 2007). Among the agents isolated from UTI in sows, uropathogenic *Escherichia coli* is the most often described (UPEC) (Drolet 2012).

The expression of virulence-encoding genes such as P (*pap*) and S (*sfa*) fimbriae allow UPEC to bind and invade host cells of the urinary tract, while iron chelator factors (siderophores) allow UPEC to capture host iron stores (Wiles et al. 2008). The ability of these strains to produce toxins such as hemolysin (*hlyA*) and cytotoxic necrotizing factor 1 (*cnf1*) promote bacterial dissemination, releasing nutrients from the host and incapacitating immune effectors cells (Wiles et al. 2008). These toxins also have the ability to produce major tissue damage, to modulate various signaling pathways of the host, affecting a range of processes including inflammatory responses, survival of the host cell, and cytoskeleton dynamics (Wiles et al. 2008).

Strains from animal and human sources are indistinguishable by the possession of certain virulence factors, phylogenetic group or serotype, which reinforces the hypothesis that farm animals play an epidemiological role in the transmission of extra-intestinal pathogenic *E. coli* (ExPEC) to humans (Vincent et al. 2010; Manges & Johnson 2012). Another important factor in relation to bacteria isolated from food animals, especially poultry and swine are the high rates of antimicrobial resistance (Aarestrup 2005). Taking into account the facts described, the objective of this study was to determine the frequency of virulence genes related to ExPEC, analyze the genetic variability of these strains and determine antimicrobial susceptibility profile of UPEC isolates from sows in Brazil.

## 2. Materials and methods

### 2.1. Sample collection and UTI screening

Three hundred urine samples from sows of different ages (gilts, low parity, and high parity sows) of three full production cycle swine herds were analyzed. The herds, selected by their history of recurrent urinary infection, were located in different cities from Sao Paulo State (Brazil) and were populated by the same genetic lineage (Landrace, Large White, and Pietrain crossbred). Sows’ repeatedly presented vulvar discharge, reduced reproductive performance, inappetence and poor body condition. Midstream urine samples were taken using a sterile universal sample collector after spontaneous micturition in the first hour of the morning. The urine samples with characteristics suggestive of UTI based on dipstick test screening results (leukocyturia, nitrite presence, proteinuria, and pH >7.5) were selected for further analysis.

### 2.2. Bacterial strains and isolation

The urine samples (10 mL) were centrifuged at 4,000*×g* for 10 minutes and the obtained pellet was plated on MacConkey agar (Difco-BBL, Sparks, MD, USA). The agar plates were incubated under aerobic conditions for 24 hours at 37 °C. One or two colonies suggestive of *E. coli* from each positive sample were identified using biochemical tests.

Each colony of interest was maintained at −86 °C in brain-heart infusion (BHI) medium (Difco, Sparks, MD, USA) with 30% of glycerol for further analysis.

### 2.3. Determination of virulence genotype by PCR

Bacteria were cultured overnight in brain-heart infusion broth – BHI (Difco-BBL, Detroit, MI, USA) at 37 °C and DNA was purified as previously described (Boom et al. 1990). Strains were tested for the *foc*H, *pap*C, *sfa*, *afa*, *hly*A, *iuc*D, *cnf*1 genes using polymerase chain reaction (PCR) (Yamamoto et al. 1995; Krag et al. 2009).

The PCR reactions contained 20 pmoles of each primer (Invitrogen Corporation, Carlsbad, CA, USA), 1.5 mM MgCl_2_, 200 mM of dNTP, 1 U of Taq DNA polymerase (Fermentas Inc., Glen Burnie, MD, USA), 1 × PCR buffer and ultra-pure water. The amplified products were separated by electrophoresis in a 1.5% agarose gel stained with BlueGreen® (LGC Biotecnologia, São Paulo, Brazil), and identified through 100 bp DNA ladder (LGC Biotecnologia).

### 2.4. Phylogenetic grouping

All isolates were assigned to phylogenetic groups according to the method of Clermont et al. (2013). This method classifies strains to one of eight phylogenetic groups (A, B1, B2, C, D, E, F, and *Escherichia* cryptic clade I) based on the presence of three genes (*chuA*, *yjaA* and *arpA*) and a specific DNA fragment (TSPE4.C2).

### 2.5. Antibiotic susceptibility testing

Susceptibility profiles were determined using disc diffusion method according to the Clinical and Laboratory Standards Institute protocol (CLSI 2015). The antimicrobial agents tested included ampicillin (10 µg), amoxicillin/clavulanic acid (20/10 µg), cefotaxime (30 µg), cefoxitin (30 µg), ceftiofur (30 µg), sulfisoxazole (300 µg), trimethoprim–sulfamethoxazole (1.25/23.75 µg), tetracycline (30 µg), nalidixic acid (30 µg), norfloxacin (10 µg), enrofloxacin (5 µg), ciprofloxacin (5 µg), florfenicol (30 µg), spectinomycin (100 µg), streptomycin (10 µg), and gentamycin (10 µg). *Escherichia coli* ATCC 25922-reference strain was used as control.

### 2.6. Single-enzyme amplification fragment length polymorphism (SE-AFLP)

Single-enzyme amplification fragment length polymorphism was performed as previously described (McLauchlin et al. 2000). DNA fragments were detected with electrophoresis at 24 V for 26 hours in 2% agarose gel stained with BlueGreen^®^ (LGC Biotecnologia) and images were captured under UV transillumination. SE-AFLP results were analyzed using the Dice coefficient by means of Bionumerics 7.5 software (Applied Maths NV, Saint-Martens-Latem, Belgium) to generate the dendrogram. Similarity value of 90% cut-off was used to analyze the clusters generated by SE-AFLP (Van Belkum et al. 2007).

### 2.7. Statistical analyses

The significance of the results was established using either Fisher’s exact test (two-tailed) or χ^2^ with the Yates correction, as appropriate. The level for statistical significance was <0.05.

## 3. Results

A total of 98 urine samples presenting turbidity, ammoniac odor, dark yellow to brown coloration, proteinuria, presence of deposit, and/or presence of nitrite were positive to *E. coli* isolation. Were selected a total of 186 *E. coli* strains, being twenty-nine from 16 sows at herd 1, 115 strains from 60 sows at herd 2, and 42 strains from 22 sows at herd 3.

The 186 strains studied were almost equally distributed by phylogenetic groups B1, D, and E (Table 1). Only three strains (1.6%) belonged to phylogroup A and all of these strains were from herd 3. Interestingly, in herds 3 and 1, most strains were classified in group B1, which generally comprise commensal strains. These results are shown in Table 1.

**Table 1.** Distribution of 186 ExPEC strains in relation to herd and phylogenetic group.

	Prevalence N (%)			
Phylogroup	Herd 1 29 (15.6)	Herd 2 115 (61.8)	Herd 3 42 (22.6)	Total 186 (100)
A	0 (0.0)	0 (0.0)	3 (7.1)	3 (1.6)
B1	13 (44.8)	26 (22.6)	25 (59.5)	64 (34.4)
D	7 (24.1)	48 (41.7)	8 (19.1)	63 (33.9)
E	9 (31.1)	41 (35.7)	6 (14.3)	56 (30.1)

Regarding the virulence genes, most strains had genes encoding F1C fimbriae (*foc*H) (78.5%) and P fimbriae (*pap*C) (58%). The distribution of both genes in relation to the phylogenetic groups was similar (Table 2), *cnf*1 and *sfa* were present in 43 (23.2%) and 21 (11.3%) strains, and also provides even distribution by phylogenetic groups. Alpha hemolysin (*hly*A) and aerobactin (*iuc*D) had lower prevalence rates (Table 2). There was no statistical difference regarding the distribution of virulence genes in relation to phylogenetic group.

**Table 2.** Frequency of ExPEC-related virulence genes in 186 porcine UPEC strains according to phylogenetic groups.

Virulence gene^a^	Phylogenetic group N(%)				Total
	A	B1	D	E	
*focH*	0	46 (24.5)	55 (30.0)	45 (24.0)	146/186 (78.5)
*papC*	1 (0.5)	30 (16.0)	42 (22.5)	35 (18.8)	108/186 (58.0)
*cnf1*	0	7 (3,8)	16 (8.6)	20 (10.6)	43/186 (23.2)
*sfa*	0	5 (2.6)	8 (4.3)	8 (4.3)	21/186 (11.3)
*hlyA*	0	2 (1.0)	0 (0.0)	1 (0.5)	3/186 (1.6)
*iucD*	0	2 (1.0)	(3.3)	1 (0.5)	13/186 (6.9)
*afa*	0	3 (1.6)	11 (6.0)	11 (6.0)	25/186 (13.4)

a The studied genes encode the following virulence factors: *afa* = afimbrial adhesin; *cnf1* = cytotoxic necrotizing factor; *focH* = F1C fimbriae subunit; *hlyA* = alpha hemolysin; *iucD* = aerobactin; *papC* = P fimbriae; *sfa* = S fimbriae.

It was observed that 182 isolates (98%) had multidrug resistance phenotype (resistant to ≥1 agent in ≥3 antimicrobial classes). Only one strain was susceptible to all antibiotics. Resistance to ampicillin was found in 149 strains (80.1%), while resistance to amoxicillin/clavulanic acid was observed in only two strains (1.1%), and the cephalosporins of second and third generation had resistance ratios between 2.6% and 0% (Table 3). Fluoroquinolones showed resistance rates between 21.5% and 33.3%. However, resistance to nalidixic acid was 66.1%. The sulfonamide, tetracycline and florfenicol resistance rates were the highest in this study, representing 94.6%, 91.9%, and 83.3% of strains, respectively (Table 3). Among the aminoglycosides, the streptomycin resistance was the higher (52.5%), gentamicin and spectinomycin resistance showed lower levels (2.8% and 11.2%, respectively).

**Table 3.** Frequency of resistance among 186 UPEC strains isolated from swine in relation to antimicrobial resistance phenotype.

Antibiotic	No.	%
Ampicillin	149	80.1
Amoxicillin/clavulanic acid	2	1.1
Cefotaxime	0	0
Cefoxitin	2	1.1
Ceftiofur	5	2.6
Sulfonamides	176	94.6
Trimethoprim-sulfamet	111	59.6
Tetracycline	171	91.9
Nalidixic acid	123	66.1
Norfloxacin	40	21.5
Enrofloxacin	62	33.3
Ciprofloxacin	42	22.5
Florfenicol	155	83.3
Spectinomycin	21	11.2
Streptomycin	98	52.6
Gentamycin	5	2.6
Susceptible to all	1	0.5
Resistant to 1 to 3 ATB	8	4.4
Resistant to 4 to 6 ATB	109	58.6
Resistant to 7 to 8 ATB	38	20.4
Resistant to 9 to 12 ATB	30	16.1

The virulence and phylogenetic groups were also evaluated for fluoroquinolones resistance status. Among the 122 susceptible strains (FQ-S) and 64 resistant (FQ-R) to ENR and/or CIP and/or NOR, the FQ-S strains showed statistical differences in the prevalence of *pap*C gene, which was positively associated with strains FQ-S. However, the plasmid gene *iuc*D was positively associated with FQ-R strains. Regarding the phylogenetic groups, there was no statistical significance (Table 4).

**Table 4.** Distribution of 186 ExPEC strains in relation to virulence traits and fluoroquinolone resistance status.

Trait	Fluoroquinolone resistance		*p*-value
	FQ-S (122)	FQ-R (64)	
Virulence			
*focH*	95	51	0.8523
*papC*	83	25	0.0002
*sfa*	13	8	0.8080
*afa*	18	7	0.5082
*hlyA*	3	0	0.5523
*iucD*	3	10	0.0015
*cnf*	24	19	0.1443
VG ≥2	78	39	0.3163
VG <2	44	25	
VG ≥3	32	15	0.7254
VG <3	90	49	
Phylogroup			
A	3	0	0.5523
B1	45	20	0.5182
E	32	23	0.1796
D	42	21	0.8715

VG = Virulence gene.

**Figure 1.**
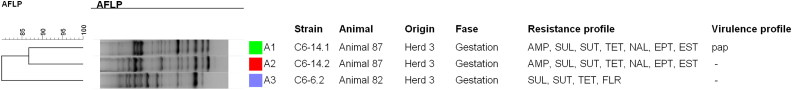
Dendrogram showing the relationship among the SE-AFLP patterns from porcine UPEC isolates from phylogenetic group A.

**Figure 2.**
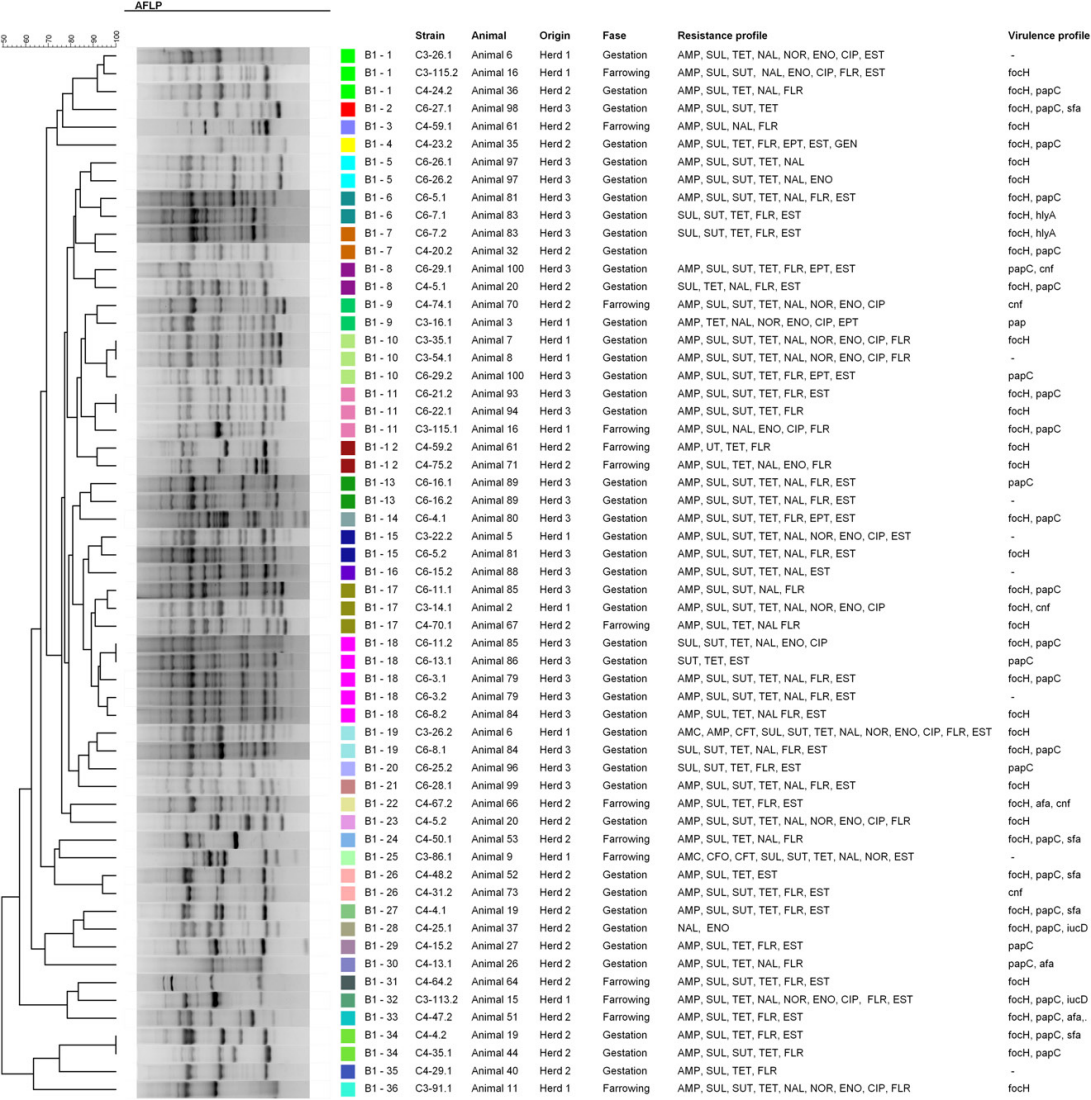
Dendrogram showing the relationship among the SE-AFLP patterns from porcine UPEC isolates from phylogenetic group B1.

**Figure 3.**
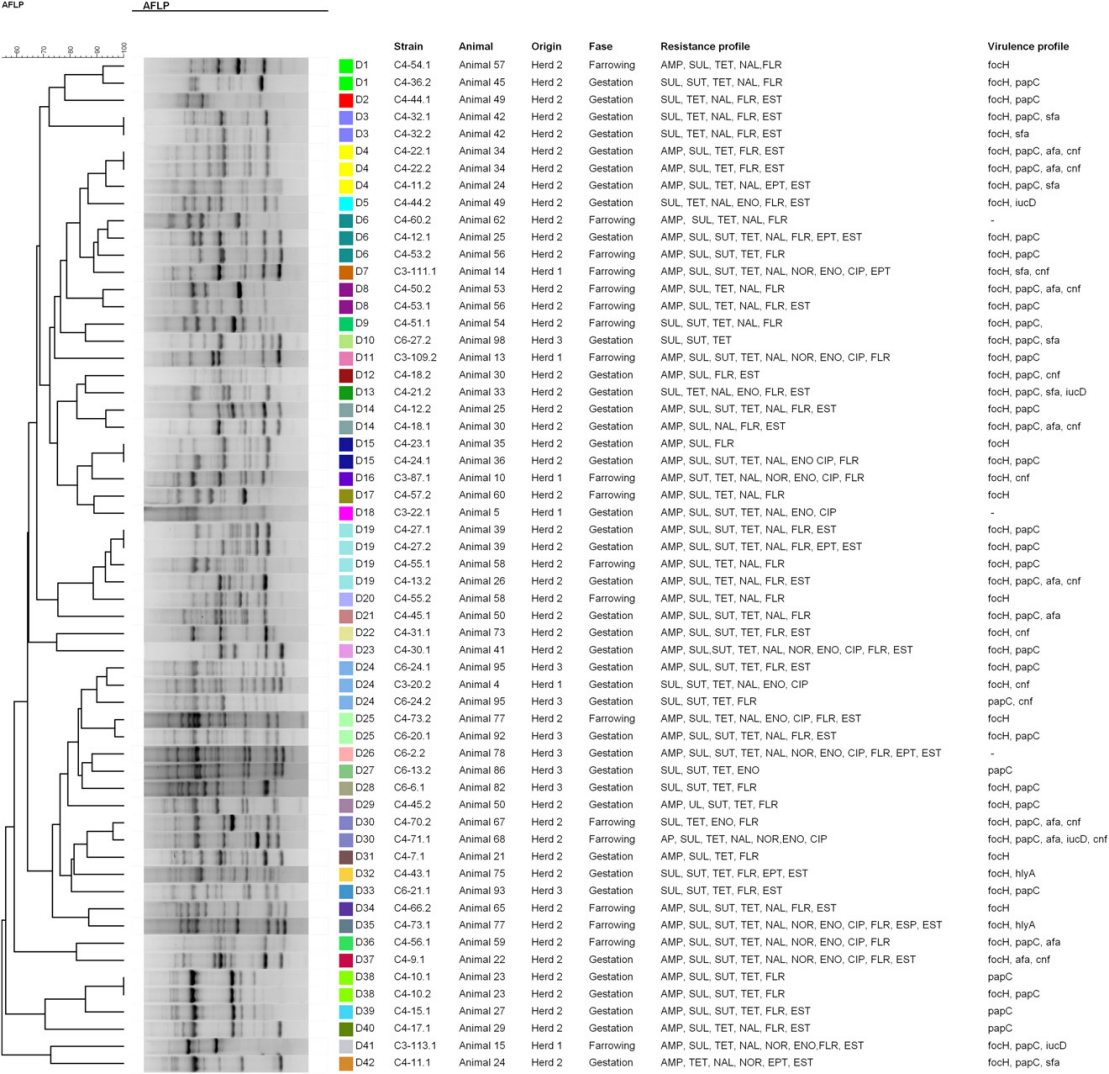
Dendrogram showing the relationship among the SE-AFLP patterns from porcine UPEC isolates from phylogenetic group D.

**Figure 4.**
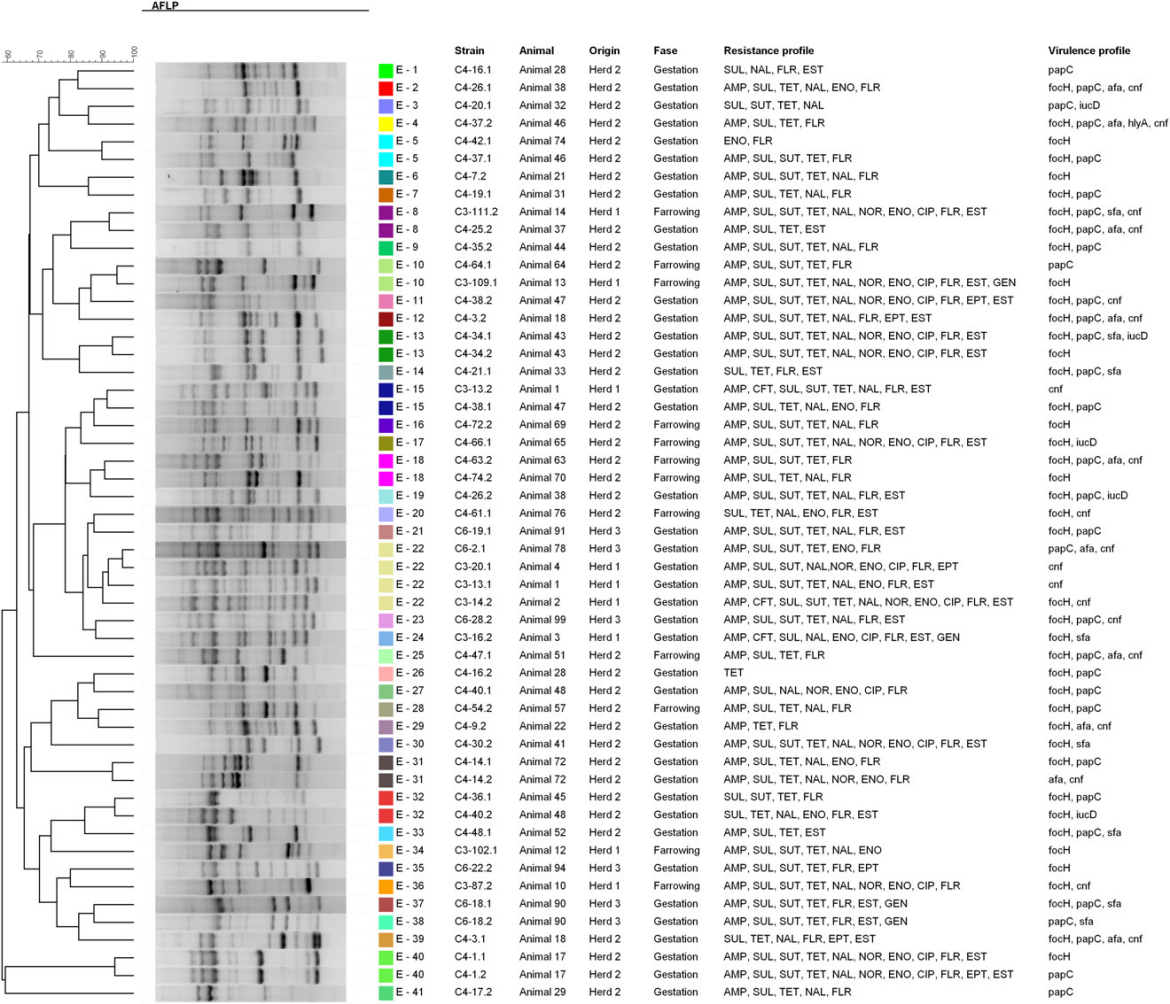
Dendrogram showing the relationship among the SE-AFLP patterns from porcine UPEC isolates from phylogenetic group E.

The clonal relationship of strains belonging to same phylogenetic group was assessed by SE-AFLP. The characterization of the strains by SE-AFLP generated three profiles in group A, 36 profiles in group B1, 42 profiles in group D and 41 in group E, with similarity equal to or greater than 90% (Figures 1–4). The strains showed 8–26 bands with size ranging from 300 bp to 10 Kb. In many cases, strains of the same animal and the same herd were grouped with 90% to 100% similarity. The correlation between resistance and SE-AFLP profiles could be observed in some groups formed.

## 4. Discussion

In swine, UTI can be found in up to 30% of intensively kept sows and is considered one of the leading causes of sudden death (Sanz et al. 2007; Kauffold et al. 2010). However, studies on the molecular epidemiology of UTI strains isolated in pigs are scarce.

In our study, 33.9% (63/186) belonged to phylogenetic groups D, group related to ExPEC strains isolated from humans. A total of 30.1% of tested strains were classified as group E that was not described in swine before, but some authors describes that this group can be formed by potential ExPEC strains and were re-assigned from the other potential ExPEC groups, D and B2 from the first phylogenetic protocol (Schmidt et al. 2015). Strains belonging to groups A and B1 had a prevalence of 1.6% and 34.4%, respectively. Although these groups are associated with commensal strains, Maynard et al. (2004) showed that a large part of ExPEC isolated from animals belonging to phylogenetic groups A and B1. These same authors also concluded that the ExPEC strains from animals showed resistance patterns and more heterogeneous phylogenetic groups, while human strains showed a homogeneous pattern. The study of Krag et al. (2009) showed that all the strains isolated from kidneys of sows with pyelonephritis lesions in Denmark belonged to groups A and B1. In a study of ExPEC isolated from pigs in China, most of the strains were classified into groups A, B1, and D (Ding et al. 2012).

In this study, we evaluated four adhesin genes that codified three fimbrial adhesins (*pap*, *sfa* and *foc*) and one afimbrial adhesin (*afa*). These adhesins play an important role in colonization and ascension to the bladder and kidneys. The highest frequency was the *foc*H gene (78.5%), which encodes a subunit of F1C fimbriae. Interestingly, in other study (Krag et al. 2009), none of the 20 strains isolated from pyelonephritis in sows was positive for F1C. This fimbriae plays an important role in the development of biofilms in biotics and abiotics surfaces and on gut colonization (Lasaro et al. 2009).

In this study, *pap*C gene was found in 108 strains (58%) and was present in strains of all phylogenetic groups. These results are very similar to described in *E. coli* isolated from urine of pigs with bacteriuria in Brazil and pyelonephritis in sows in Denmark, respectively (De Brito et al. 1999; Krag et al. 2009). In both studies, the prevalence of P fimbriae was 58.4% and 50%, respectively. S fimbriae (*sfa*) are commonly found in ExPEC strains from cystitis, suggesting that the expression of *sfa* is a selective advantage in the lower urinary tract. The prevalence 11.3% (21/186) corroborates with the findings in ExPEC strains in humans and animals, including pigs, demonstrating the prevalence of *sfa* (not associated with *foc*) between 4% and 11% (Johnson & Stell 2000; Johnson et al. 2003; Ding et al. 2012; Tan et al. 2012).

The gene marker of aerobactin *iucD* was found in 13 strains (6.9%). Despite the fact that operon of aerobactin can occur in UPEC strains, it is strongly associated with plasmids found in ExPEC strains pathogenic to birds (Avian pathogenic Escherichia coli (APEC)) (Tivendale et al. 2004; Mellata 2013).

Two toxins are usually found in UPEC: cytotoxic necrotizing factor 1 (*cnf1*) and α-hemolysin (*hly*A) (Smith et al. 2007; Wiles et al. 2008). The α-hemolysin is encoded by ∼50% of UPEC strains from humans and its expression is associated with increased clinical severity in UTI patients (Johnson & Russo 2005). In this study, *hly*A was present in only three strains (1.6%), but compared to other studies, the prevalence of this gene in ExPEC is varied. In study carried out in China with ExPEC strains from pigs, *hly*A prevalence was 17.8% (Tan et al. 2012). The gene *cnf*1 encodes a cytotoxic necrotizing factor that increases the resistance of bacterial cells to the attack of neutrophils (Smith et al. 2007). About a third of human UPEC strains have the gene *cnf*1, including the prototypical strain UTI89 (Wiles et al. 2008). The presence of 41 strains (23.2%) in our strains corroborate these findings.

Studies on the association of resistance and virulence suggest that resistance to (fluoro) quinolones may be associated with a decrease in the presence of some virulence factors, such as *pap*, *sfa*, *cnf*1, *hly*A, *iuc*D in ExPEC (Johnson et al. 2003; da Silva & Mendonça 2012; Giufrè et al. 2012). These studies have concluded that strains resistant to (fluoro) quinolones show less virulence genes and are less associated with phylogrupo B2, group that present a greater number of virulence-associated genes (Johnson et al. 2003; da Silva & Mendonça 2012; Giufrè et al. 2012). Our study showed that the strains susceptible to fluoroquinolones (FQ-S) were positively associated with the gene *pap*C (*p* = 0.0002), data that corroborate different studies (Vila et al. 2002; Johnson et al. 2003). However, in their studies, the strains containing genes *cnf*1, *sfa*, and *hly*A also had a positive association with the FQ-S status, different from our findings, where these genes showed no statistical correlation with FQ-S status. In contrast, in our study the gene encoding a subunit of aerobactin, *iuc*D, was positively associated with strains resistant to fluoroquinolones (FQ-R) (*p* = 0.0015). Of all the genes studied by us, the only one that is located on plasmids is *iuc*D. This may be because the resistance status to fluoroquinolones is mainly associated with virulence factors encoded in chromosomal pathogenicity islands and phylogenetic group.

In pig production systems worldwide, large amounts of antimicrobial agents are used for therapy and disease prophylaxis (Aarestrup 2005). The fact that 98% (182/186) of strains presented multidrug resistance phenotype and only one was susceptible to all antibiotics is alarming.

In Europe, the level of *E. coli* resistant to nalidixic acid recovered from pig production is low (Garcia-Migura et al. 2014), which differs widely from our study that shows a resistance rate to nalidixic acid of 66.1%. In Brazilian UPEC strains, resistance rates of enrofloxacin, norfloxacin, and ciprofloxacin were 33.3%, 21.5%, 22.5%, respectively. These rates of second generation fluoroquinolones resistance are lower than those presented by other authors (Jiang et al. 2011; Tang et al. 2011) in ExPEC isolated from pigs in China (between 50% and 82.2%) and close to the quinolone resistance level found in *E. coli* isolated from pigs in South Korea (Lee et al. 2014). There are no published data on the prevalence of quinolone resistance in ExPEC pigs in Brazil, but a similar rate of ExPEC quinolone resistant strains are described in Brazilian commercial turkeys (Cunha et al. 2014).

In relation to beta-lactams, resistance to ampicillin was high (80.1%); however, amoxicillin, and clavulanic acid and second and third generation cephalosporins presented low rates, ranging from 0% to 2.6%, data that corroborate the findings in *E. coli* isolated in five pig farms in Canada (Kozak et al. 2009). High rates of resistance to aminopenicillins are described in several studies carried out with *E. coli* isolated from pigs in Asia and North America (Kozak et al. 2009; Jiang et al. 2011; Malik et al. 2011; Tang et al. 2011; Tadesse et al. 2012; Lee et al. 2014) and varies among European countries (Garcia-Migura et al. 2014).

The high prevalence of strains resistant to florfenicol in our study is in accordance with several studies in pathogenic and commensal *E. coli* from pigs (Jiang et al. 2011; Wang et al. 2011). Comparing strains from pigs with ExPEC strains isolated from other food-producing animals, such as poultry, it is observed that the latter tend to have a higher susceptibility to florfenicol (Jiang et al. 2011; Cunha et al. 2014). The resistance to florfenicol is mediated by *flo*R gene, which is widely distributed in diseased or healthy pigs (Maynard et al. 2004; Wang et al. 2011). In Brazil, this antibiotic is widely used for the treatment of reproductive and urinary infections in sows and respiratory diseases in growing-finishing pigs.

To assess genetic diversity, strains from the same phylogenetic group were subjected to SE-AFLP analysis. Multiple AFLP profiles were found, which shows a large genetic diversity of UPEC isolate from sows. Most strains were grouped according animal and herd of origin.

In conclusion, our findings indicate that the population of UPEC strains isolated from sows in Brazil presented a different repertoire of virulence, with the prevalence of virulence genes in common with human and animal ExPEC. The strains were predominantly classified into groups B1, D and E and exhibit a multidrug-resistance phenotype that could be associated with the indiscriminate use of these drugs in pig production.
